# Analysis and Modeling of Air Pollution in Extreme Meteorological Conditions: A Case Study of Jeddah, the Kingdom of Saudi Arabia

**DOI:** 10.3390/toxics10070376

**Published:** 2022-07-05

**Authors:** Mohammad Rehan, Said Munir

**Affiliations:** 1 Center of Excellence in Environmental Studies (CEES), King Abdulaziz University, Jeddah 21589, Saudi Arabia; 2 Institute for Transport Studies, Faculty of Environment, University of Leeds, Leeds LS2 9JT, UK; s.munir@leeds.ac.uk

**Keywords:** extreme value analysis, quantile regression, air pollution, ozone, nitrogen oxides, supervised machine learning, climate change

## Abstract

Air pollution has serious environmental and human health-related consequences; however, little work seems to be undertaken to address the harms in Middle Eastern countries, including Saudi Arabia. We installed a continuous air quality monitoring station in Jeddah, Saudi Arabia and monitored several air pollutants and meteorological parameters over a 2-year period (2018–2019). Here, we developed two supervised machine learning models, known as quantile regression models, to analyze the whole distribution of the modeled pollutants, not only the mean values. Two pollutants, namely NO_2_ and O_3_, were modeled by dividing their concentrations into several quantiles (0.05, 0.25, 0.50, 0.75, and 0.95) and the effect of several pollutants and meteorological variables was analyzed on each quantile. The effect of the explanatory variables changed at different segments of the distribution of NO_2_ and O_3_ concentrations. For instance, for the modeling of O_3_, the coefficients of wind speed at quantiles 0.05, 0.25, 0.5, 0.75, and 0.95 were 1.40, 2.15, 2.34, 2.31, and 1.56, respectively. Correlation coefficients of 0.91 and 0.92 and RMSE values of 14.41 and 8.96, which are calculated for the cross-validated models of NO_2_ and O_3_, showed an acceptable model performance. Quantile analysis aids in better understanding the behavior of air pollution and how it interacts with the influencing factors.

## 1. Introduction

Air pollution has emerged as a serious and growing environmental issue affecting human health, natural environment, biodiversity, building materials, and visibility. The increasing urbanization, population, and consumption of fossil fuels for energy and transportation needs have resulted in significant increase in air pollution [1,2]. In 2015, air pollution resulted in 6.4 million deaths worldwide [3]. Moreover, air pollution is known to cause several respiratory diseases, cardiovascular problems, lung cancer, and asthma [4]. Long-term exposure to elevated levels of particulate matter and nitrogen dioxide (NO_2_) may cause cardiovascular problems and lung cancer, resulting in premature death and hospital admission [5]. It is reported that children, elderly, and people with long-term health problems are more vulnerable to the negative effect of air pollution [6]. The adverse impacts of air pollution are associated with the duration of exposure and levels of air pollutants, and higher levels and long-term exposure cause more severe negative impacts. Exposure to elevated levels of ground level ozone affects the respiratory and cardiovascular system [7]. Furthermore, ozone increases DNA damage in epidermal keratinocytes and leads to impaired cellular function [8]. For more details on the health effects of air pollution, see [7,8,9,10,11,12]. In addition to human health, air pollution reduces crop yield, affects the quality of fresh produce, and damages monuments and historical buildings [6,13].

The main factors influencing air pollution are emission sources, meteorological conditions, and geographical characteristic conditions [14]. In large urban cities, the main emission sources of air pollutants are road traffics; however, point sources (e.g., industrial emissions) and area sources (e.g., emissions from houses and other minor sources) also contribute significantly to total emissions [15]. Atmospheric concentrations of air pollutants are directly related to local emissions of air pollutants from various sources [16]; however, urban and regional scale emissions also contribute significantly to background concentrations [17]. In regression models, the background concentration is represented by the intercept of the model, whereas in dispersion models, it is directly added to the modeled concentrations. Meteorological conditions, in addition to emission sources, play a vital role in the dispersion and chemical transformation of pollutants; however, the effect of meteorology is not straightforward and is compounded by local geographical conditions and the type of pollution [18]. For example, the higher wind speed quickly disperses locally emitted pollutants, but it may bring emissions from upwind areas. Similarly, high temperature and solar radiation help in encouraging both vertical and horizontal dispersion of pollutants; however, at the same time, this help in photochemical O_3_ formation. Furthermore, the effect of temperature may vary at different levels of pollutant concentrations, which will be explored further in this paper. For more details on the variable effect of temperature on the ozone, see [19,20,21].

To successfully manage and control air pollution, it is important to first characterize air quality in terms of air pollutant levels, spatial and temporal trends, and interaction with other driving factors. For this purpose, air quality monitoring and modeling are the two most important tools. Air quality monitoring informs us about the current status of air pollution, whereas modeling helps us in predicting the future status of air pollutants. Furthermore, air quality modeling is carried out to investigate how air pollution impacts human health, how pollutants interact with each other and with weather conditions, how different emission scenarios affect future pollution levels, how pollutants are dispersed and transformed in the atmosphere, how to quantify their long-term temporal trends, and finally, how to fill spatiotemporal gaps in monitored data [22,23,24,25,26,27,28,29,30,31]. Mainly, three approaches are used for air quality modeling, including: (1) Dispersion models, such as ADMS-Urban and Airviro, (2) statistical and machine learning models, such as multiple linear regression models, generalized additive models, random forest, and quantile regression models, and (3) chemical-dynamical models, such as WRF-Chem, GEOS-Chem, CMAQ, and CAMx. In this paper, a supervised machine learning technique, known as quantile regression model, has been employed to analyze the whole distribution of the modeled variable, rather than only focusing on the mean value as compared with the multiple linear regression.

Jeddah is the second largest city of the Kingdom of Saudi Arabia, with a population of around 3.5 million. It is a coastal city located in the middle of the eastern coast of the Red Sea, known as the Bride of the Red Sea, and is considered the economic and tourism capital of the country. Jeddah is the fourth largest industrial city in Saudi Arabia with a dense transport infrastructure network [32,33]. Recently, several research studies have been published on the levels of different air pollutants, mostly particulate matter, in Jeddah. For example, the authors of [34] studied fine and coarse particulate matter sources in Jeddah and reported higher levels of PM_2.5_ (21.9 μg/m^3^) and PM_10_ (107.8 μg/m^3^), which exceeded the WHO guidelines for PM_2.5_ (10 μg/m^3^) and PM_10_ (20 μg/m^3^). Another study analyzed particulate matter and number concentrations of particles larger than 0.25 µm in the urban atmosphere of Jeddah [35]. Khodeir et al. [36] studied source apportionment and elemental composition of PM_2.5_ and PM_10_ in Jeddah and reported overall mean concentration of 28.4 ± 25.4 μg m^−3^ for PM_2.5_ and 87.3 ± 47.3 μg m^−3^ for PM_10_, with significant temporal and spatial variability. Khodeir et al. [36] only focused on PM and did not analyze the levels of NO_2_ and O_3_ in their study. Porter et al. [37] analyzed the levels of O_3_, NO_2_, and PM_10_ and studied their association with meteorological data in Jeddah. Similar to the current study, in Porter et al. [37], oxides of nitrogen (NO, NO_2_, and NO_x_) were measured using chemiluminescent detectors (Environment S.A. France AC32M), O_3_ was measured via ultraviolet absorption using O_3_42 module ozone analyzers, and PM_10_ was measured by beta gauge (Environment S.A. MP101M). The authors used only descriptive statistics and graphical presentations in their study and did not use any modeling approaches.

Several authors reported low levels of O_3_ in Jeddah and stressed the need for further assessments. Similarly, Hassan et al. [33] investigated the levels of ambient O_3_ and NO_2_ along with meteorological data in Jeddah. They found O_3_ to be highly dependent on the NO_x_ diurnal cycle and wind speed. Furthermore, they reported that NO_x_ exceeded WHO air quality standards, especially in industrial sites. Few other studies reported air pollution data from the city of Makkah, Saudi Arabia with similar weather conditions [28,38,39]. However, none of these studies carried out advanced modeling, especially employing the quantile regression model for NO_2_ and O_3_ to analyze how their whole distribution is associated with other pollutants and meteorological conditions in Jeddah.

Th present study focused on the analysis and modeling of ambient air pollution in extreme meteorological conditions in Jeddah, the Kingdom of Saudi Arabia. For this purpose, a continuous air quality monitoring station (AQMS) was designed and fabricated at a local factory in Jeddah and deployed close to the Center of Excellence in Environmental Studies at King Abdulaziz University, Jeddah. Several air pollutants (NO, NO_2_, NO_x_, O_3_, CO, SO_2_) and meteorological parameters, namely wind speed (WS), wind direction (WD), temperature (Temp), and relative humidity (RH), were measured during 2018 and 2019. Finally, a supervised machine learning model known as quantile regression model was developed in R programming language [40] and its package ‘quantreg’ [41]. NO_2_ and O_3_, two of the most important gaseous pollutants from a public health perspective, were analyzed to study their relationship with other gaseous pollutants and meteorological parameters in Jeddah, focusing on the extreme values of the distribution of the modeled variable. Unlike most of the current literature which focuses on the mean values, this study diverts the focus to the whole distribution of the response variable, especially to the left and right tails of the distribution, which are more important from a public health perspective.

## 2. Materials and Methods

### 2.1. Air Quality and Meteorological Data

**Figure 1:**
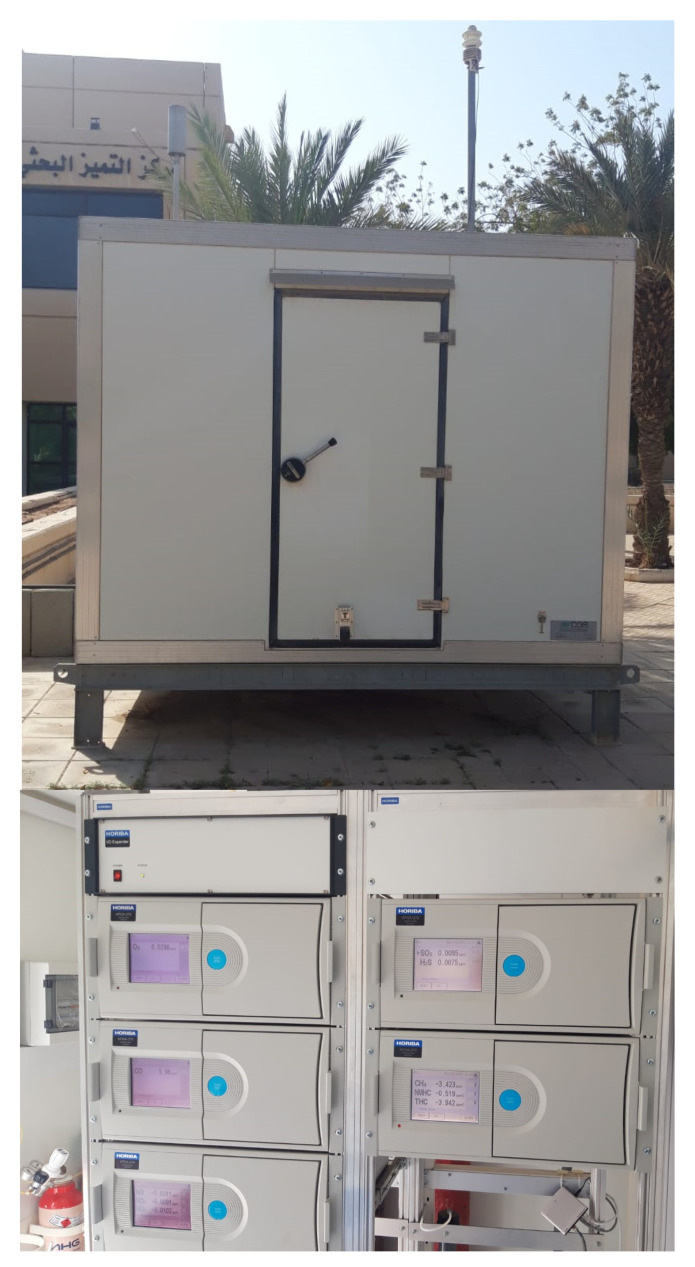
Air quality monitoring station (AQMS): Outside (**top**) and inside (**bottom**) view.

**Figure 2:**
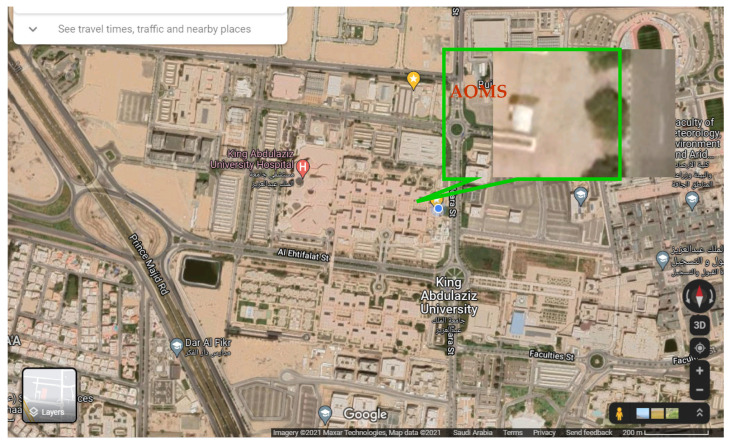
Map of the location of air quality monitoring station (AQMS) situated within King Abdulaziz University, Jeddah, Saudi Arabia.

A continuous air quality monitoring station (AQMS) was designed and fabricated in a local workshop in Jeddah, the Kingdom of Saudi Arabia (Figure 1). Five different gas analyzers were purchased from Horiba (Kyoto, Japan) and installed in the station. These included top-of-the range systems, namely APNA-370 (NO_2_, NO, NO_x_), APSA-370 (SO_2_, H_2_S), APOA-370 (O_3_), APHA-370 (THC, NMHC, CH_4_), and APMA-370 (CO). The calibration gases with high purity were purchased from a local supplier in Jeddah. The AQMS was deployed close to the Center of Excellence in Environmental Studies at King Abdulaziz University, Jeddah, which was used to collect hourly pollutant concentrations during 2018 and 2019. The station is located a few meters from an internal university road, but away from major congested roads. Moreover, traffic on this internal road significantly reduces during evenings, weekends, and academic vacations, and is mostly limited to university staff. Several pollutants were monitored, namely nitric oxide (NO), nitrogen dioxide (NO_2_), nitrogen oxide (NO_x_), ozone (O_3_), carbon monoxide (CO), and sulphur dioxide (SO_2_). A map of the monitoring site is shown in Figure 2. Furthermore, a weather station was installed on top of the AQMS to measure meteorological parameters, including wind speed (WS), wind direction (WD), temperature (Temp), and relative humidity (RH). An AC was installed within the AQMS to maintain the inside temperature between 24–28 °C, which could otherwise increase to above 50 °C during the hot summer, with risks of damaging the installed systems. The gas analyzers were calibrated on a regular basis to achieve high quality data.

The data have been collected on a continuous basis in an hourly resolution and recorded on a data logger installed inside the station, which is then transferred to a PC as required through a modem. Pollutants were expressed in parts per billion (ppb), except for CO, which was expressed in parts per million (ppm). Temperature was expressed in degree Celsius (°C), relative humidity in percentage (%), wind speed in meter per second (m/s), and wind direction in degrees from the north (°N). The AQMS used the reference techniques to measure the pollutant concentrations, using chemiluminescence analyzer for the monitoring of NO_x_, NO_2_, and NO, UV fluorescence analyzer for the monitoring of SO_2_, UV absorption analyzer for the monitoring of O_3_, and infra-red absorption analyzer for the monitoring of CO. The systems’ specifications, including measurement principles and detection limits, are provided in Table 1.

### 2.2. Statistical Analysis

This paper employed a quantile regression approach for the modeling of O_3_ and NO_2_ using several predictors of gaseous pollutants and meteorological parameters (Equations (1) and (2)). Multiple linear regression specifies the conditional mean function, which analyzes the effect of covariates (predictors) on the mean of the response variable, whereas quantile regression specifies the conditional quantile function. This indicates that quantile regression allows the covariates to have different effects at different quantiles of the response variable distribution. For more details on quantile regression, see [25,28,43,44]. To assess the model performance, the data were divided into randomly selected training (75%) and testing dataset (25%). The model was fitted on the training and cross-validated on the testing data. As reported by [25], quantile regression is robust (insensitive) to departures from normality and to skewed tails. In this paper, two models were developed for the modeling of O_3_ (Equation (1)) and NO_2_ (Equation (2)).

O_3_ = β_o_^(p)^ + β_1_^(p)^NO + β_2_^(p)^NO_2_ + β_3_^(p)^CO + β_4_^(p)^SO_2_ + β_5_^(p)^WS + β_6_^(p)^WD + β_7_^(p)^RH + β_8_^(p)^Temp + ε_i_ (1)

NO_2_ = β_o_^(p)^ + β_1_^(p)^NO + β_2_^(p)^O_3_ + β_3_^(p)^CO + β_4_^(p)^SO_2_ + β_5_^(p)^WS + β_6_^(p)^WD + β_7_^(p)^RH + β_8_^(p)^Temp + ε_i_ (2)

In Equations (1) and (2), βo represents the intercept, β1 to β8 represent coefficients (slopes) of the covariates, and ε shows the error terms of the models, which is the difference between the modeled and measured concentrations. The (p) shows the p-th quantile, and its value lies between 0 and 1. In this study, we used five quantiles 0.05, 0.25 (first quartile), 0.5 (median), 0.75 (third quartile), and 0.95. To assess the model performance, several statistical metrics were used, including correlation coefficient (r), coefficient of determination (R^2^), root mean squared error (RMSE), and factor of two (FAC2). For more details regarding these metrics and how to calculate them, see [45,46].

The R programming language [40] and the packages ‘openair’ [47] and ‘quantreg’ [41] were used to develop the models, perform general statistical data analysis, and develop visualizations.

## 3. Results and Discussion

**Figure 3:**
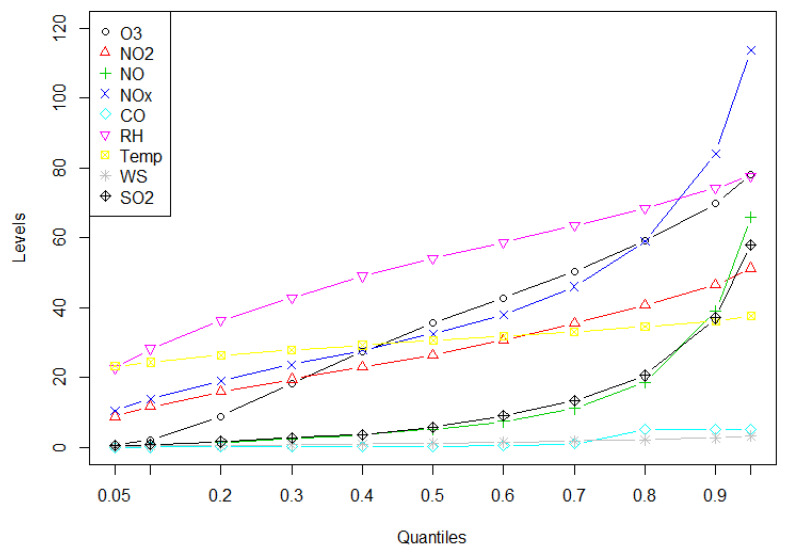
Levels of various air pollutants and meteorological parameters at different quantiles of distributions. All of the pollutants are measured in ppb, except for CO, which was measured in ppm. In the figure, WS stands for wind speed (m/s), WD for wind direction (degree from the north), Temp for temperature (°C), and RH for relative humidity (%).

A summary of the data is provided in Table 2 and more details are depicted in Figure 3, where levels of various variables are shown at different quantiles (0.05, 0.1, 0.2, 0.3, 0.4, 0.5, 0.6, 0.7, 0.8, 0.9, and 0.95) of their distribution.

NO_2_, SO_2_, O_3_, and CO are important pollutants from human health and environmental perspectives. CO, SO_2_, and NO_2_ are combustion-related pollutants, mainly emitted by the combustion of fossil fuels used in road traffic and different industries. O_3_ is predominantly a secondary pollutant, formed in the atmosphere by the photochemical reaction of its precursors in the presence of solar radiation. These pollutants have the strongest evidence for public health concern. Health problems can occur as a result of both short- and long-term exposure to these pollutants [48]. Exposure to the elevated levels of ozone can cause problems in breathing, trigger asthma, reduce lung function, and lead to lung disease. NO_2_ and SO_2_ are related to asthma and other respiratory conditions. CO, once inhaled, diffuses into the lung tissues and bloodstreams, which affect the oxygen levels of blood resulting in tissue and cell damage [48,49,50].

**Figure 4:**
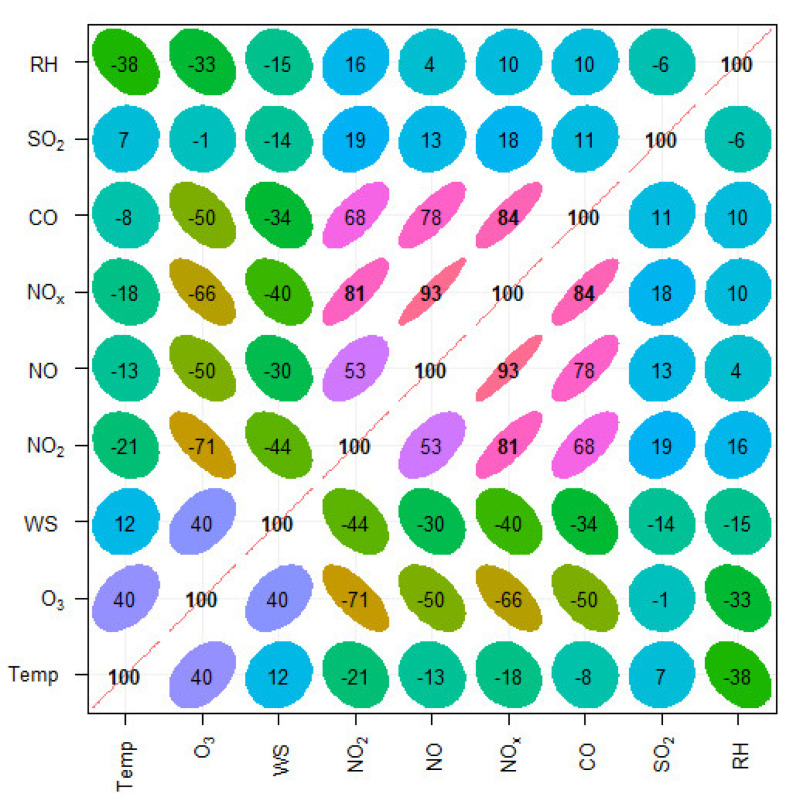
Correlation between various air pollutants and meteorological parameters. Decimal point is not shown to facilitate presentation (e.g., 0.93 is shown as 93).

The results of simple correlation between different air pollutants and meteorological parameters are shown in Figure 4, which shows how various variables are related to each other. Figure 4 shows that combustion-related species, such as NO, NO_2_, NO_x_, and CO, are strongly positively correlated with each other. These pollutants are predominantly emitted by road traffic. Their correlation coefficient (r-values) range from 0.53 to 0.93. These species are clustered in the middle of the plot. SO_2_ is also a combustion-related pollutant, but is mostly emitted by industrial activity, which processes materials that contain sulphur, mainly from the combustion of coal and oil-containing sulphur. In the past, road traffic was considered a major source of SO_2_, but since the desulphurization of vehicle fuels, this is not the case anymore. Therefore, SO_2_ has shown weak correlation with NO_x_ and CO species (Figure 4). O_3_ is strongly negatively correlated with traffic-related air pollutants and the r-values between O_3_ and traffic-related pollutants range from −0.50 for CO to −0.71 for NO_2_. Negative correlation between O_3_ and these species is well known [28,51,52]. O_3_ is a secondary air pollutant and predominantly produced in the atmosphere by the photochemical reaction of its precursors, such as NO_x_ and hydrocarbons, in the presence of solar radiation. This is possibly the reason that O_3_ is positively correlated with meteorological parameters, especially temperature and solar radiation. In contrast, high wind speed and high temperature help in the dispersion of locally emitted air pollutants (e.g., NO_x_ and CO), which explains the negative association between them.

The problem with correlation analysis is that it shows only a linear relationship between the two variables, due to the fact that the relationship of pollutants with other pollutants and with meteorological parameters is not always linear and may change at different levels of the variables. Therefore, correlation analysis or simple linear regression cannot fully describe the association between different variables. Therefore, an advance approach is required to analyze the association at various levels of the variables. A quantile regression approach is used to view how the relationship of explanatory variables (covariates) changes at different levels of the response variables (modeled pollutants), as shown in Figure 4.

**Figure 5:**
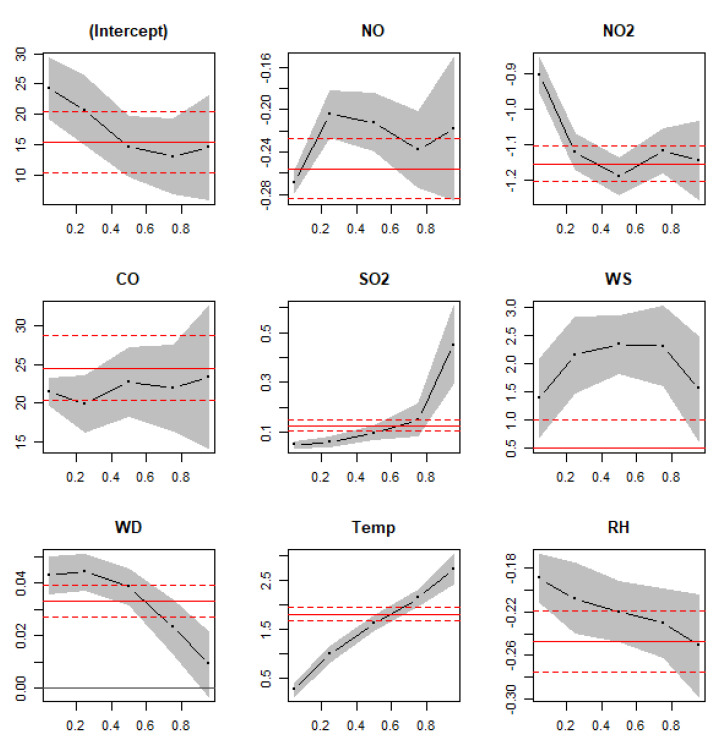
Outputs of the quantile regression model for analyzing O_3_ concentrations (ppb) using several explanatory variables in Jeddah, Saudi Arabia, 2018–2019. The black dotted dashed lines show coefficients at different quantiles of the covariates and the shaded areas show the confidence intervals. The red solid lines show the coefficients of ordinary multiple linear regression model, whereas the red dotted lines show the confidence intervals of the mean coefficients. *x*-axes show quantiles of the dependent variable and *y*-axes show the regression coefficients for specified independent variables.

Figure 5 shows the output of a quantile regression model using O_3_ as a response variable and NO, NO_2_, CO, SO_2_, Temp, RH, WD, and WS as explanatory variables (also known as predictors or covariates). The values on *x*-axis show different quantiles used in the model (0.05, 0.25, 0.50, 0.75, 0.95), whereas the values on *y*-axis show coefficients of the predictors. Positive coefficients indicate a positive association, whereas negative coefficients show a negative association between the response (modeled) and explanatory variables. Furthermore, the larger the values of coefficients, the stronger the effect of the explanatory variable on the response variable. Figure 5 shows that NO, NO_2_, and RH have a negative association with O_3_, whereas the rest of the parameters have a positive association with O_3_. It is shown (Figure 5) that values of the coefficients change at different quantiles of the distributions. The red solid line is the mean line, which can be considered as the coefficient of the ordinary least square model. Grey area around the black line and dashed red lines around the solid-red line show the confidence intervals of the quantile coefficients and mean coefficient, respectively. It should be noted that the effect of all covariates (predictors) at all quantiles is significant as the confidence intervals do not overlap with the zero line. When the confidence intervals of mean coefficient overlap with the confidence intervals of any quantile, this indicates that the mean effect is not significantly different from the quantile effect. NO_2_ has a significantly different effect from the mean effect at quantile 0.05, whereas SO_2_ has a significantly different effect on quantiles 0.05, 0.1, and 0.95. Both WS and Temp have a significantly different effect from the mean effect. The effect of CO is not significantly different from the mean effect at any quantile. WS, Temp, and CO have larger coefficient values, and thus, have a stronger association with the modeled variable. The coefficients in Figure 5 are presented in Table 3.

**Figure 6:**
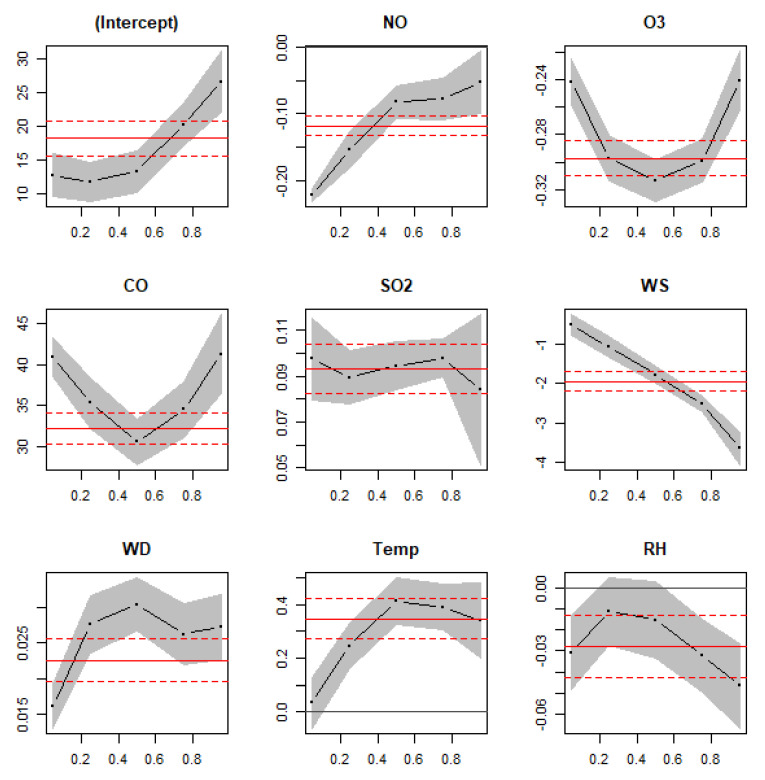
Outputs of quantile regression model analyzing NO_2_ concentrations (ppb) using several explanatory variables in Jeddah, Saudi Arabia, 2018–2019. The black dotted dashed lines show coefficients at different quantiles of the covariates and the shaded areas show the confidence intervals. The red solid lines show the coefficients of ordinary multiple linear regression model, whereas the red dotted lines show the confidence intervals of the mean coefficients. *x*-axes show quantiles of the dependent variable and *y*-axes show the regression coefficients for specified independent variables.

Figure 6 shows the outputs of quantile regression model using NO_2_ as a response variable and O_3_, NO, CO, SO_2_, WS, WD, RH, and Temp as explanatory variables. Figure 6 shows how the association between response and explanatory variables varies at different quantiles of the modeled variable. Coefficients of different variables at different quantiles are shown in Table 4. It is shown that the effect of all covariates at all quantiles is significant as the confidence intervals do not overlap with the zero line, except for the temperature at quantile 0.05. Ground level O_3_, NO, relative humidity, and wind speed have shown negative associations, whereas the other variables have shown a positive association with NO_2_ at all quantiles. O_3_ concentrations have shown a significantly different effect from the mean effect only at quantiles 0.05 and 0.95, whereas NO has only shown a significantly different effect from the mean effect at quantile 0.05. Moreover, CO has shown a positively significant difference from the mean effect at quantiles 0.05 and 0.95. This shows the importance of studying the effect of the predictors on both left and right tails of the distribution of the modeled variable. From a public health perspective, it is important to analyze the effect of explanatory variables on atypically high pollution levels, rather than only analyzing the mean effect.

**Figure 7:**
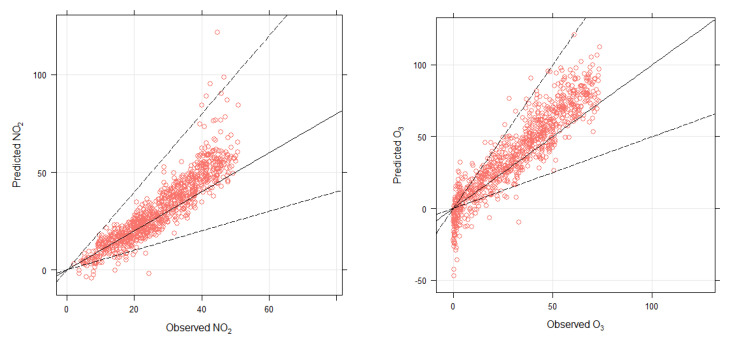
Graphical comparison of observed and predicted O_3_ concentrations (ppb) (**right**-panels) and NO_2_ concentrations (ppb) (**left**-panels) for testing data.

The performance of the two models for O_3_ (Figure 5) and NO_2_ (Figure 6) was assessed by comparing predicted and observed concentrations. Graphical comparison of predicted and observed concentrations of both NO_2_ and O_3_ is shown in Figure 7. Comparison was carried out for 25% of testing dataset (cross-validation), which was randomly selected and not included in the model fitting process. Observed and predicted concentrations showed a strong association with each other for both NO_2_ and O_3_. To quantify the relationship between observed and predicted concentrations, several statistical metrics were calculated, including correlation coefficient (r), coefficient of determination (R^2^), root mean square error (RMSE), and factor of two (FAC2). The factor of two (FAC2) is the percentage of the predictions within a factor of two of the observed values. Correlation coefficients of 0.92 and 0.91 between modeled and observed concentrations show a strong correlation between observed and predicted O_3_ and NO_2_ concentrations, respectively. Furthermore, coefficient of determination of 0.86 and 0.83 shows that with the help of the predictors used in the models, quantile regression model was able to explain 86% of the variation in O_3_ concentration and 83% of the variation in NO_2_ concentration. These statistical metrics (Table 5) indicate an acceptable model performance. Metrics calculated for multiple linear regression models are provided for comparison only, which showed that quantile regression models outperformed the multiple linear regression models. The authors of [46] modeled PM_10_ concentrations in Makkah and compared the performance of several models, including multiple linear regression, generalized additive model, and quantile regression model. They showed that the quantile regression model outperformed the other models, which justifies the use of this model for air pollution modeling. In this paper, the purpose is not to compare the performance of quantile regression model with other models, rather the aim is to show that it is important to analyze the whole distribution of the modeled variables, especially the atypically high concentrations of air pollutants, which are important from a public health perspective.

Harkey et al. [53] analyzed the relationship between several meteorological parameters and NO_2_ using ground-level and satellite-based observations employing the Environmental Protection Agency (EPA) Community Multiscale Air Quality (CMAQ) model. They found that the boundary layer height, wind speed, temperature, and relative humidity were the most important variables in determining near-surface NO_2_ variability. NO_2_ concentration was negatively associated with planetary boundary height, wind speed, and insolation, and positively associated with temperature. Wang et al. [54] analyzed the spatiotemporal trends of NO_x_ in relation to land use and meteorological factors in Accra Metropolis during April 2019 to June 2020. They found strong correlations between NO_2_ and NO_2_/NO_x_ with mixing layer depth, incident solar radiation, and water vapor mixing ratio.

Hu et al. [55] conducted a detailed analysis on ground level O_3_ and developed several generalized additive models (GAMs) to predict the maximum daily 8-h O_3_ concentration in 334 cities in China. The correlation between O_3_ and meteorological variables varied spatially; however, generally temperature, relative humidity, and sunshine hours were the most important three influencing factors for O_3_. Furthermore, Hu et al. [55] reported that the influence of these meteorological factors on O_3_ concentration was nonlinear, which agreed with our findings. The average R2 of the GAMs model for all cities was 0.72. In another study, Camalier et al. [56] collected data from 39 major eastern US urban areas and developed a generalized linear model (GLM) for modeling the effect of meteorology on ground level O_3_. The GLM model explained 80% of the variations in O_3_ and reported that O_3_ generally increased with increasing temperature and decreased with increasing relative humidity.

## 4. Conclusions

In this paper, we modeled the concentrations of ground level O_3_ and NO_2_, which are two important atmospheric pollutants from a public health perspective. Several air pollutants (CO, NO, NO_2_, NO_x_, O_3_, and SO_2_) and meteorological parameters (WS, WD, Temp, and RH) were measured in the city of Jeddah, the Kingdom of Saudi Arabia during 2018–2019. First, the correlation analysis was performed to determine the simple correlation between different pollutants and meteorological parameters. Combustion-related air pollutants (e.g., NO, NO_2_, and CO) showed a positive correlation with each and a negative correlation with O_3_, temperature, and wind speed. O_3_ was positively correlated with temperature and wind speed and negatively correlated with relative humidity. However, the simple correlation analysis is unable to present a holistic picture of the association between different pollutants and meteorological parameters. This is due to the fact that the association between different pollutants and meteorology varies at different levels of the pollutants and meteorological parameters. Quantile regression model was able to explore this association further and showed how the strength of the relationship changed at different levels of the pollutants. Ground level O_3_ and NO_2_ were modeled employing quantile regression model using several air pollutants and meteorological parameters as predictors. The models demonstrated the importance of analyzing both tails of the distribution, e.g., at quantile 0.95 and 0.05. Finally, the model performance was assessed using graphical presentation and several statistical metrics, including correlation coefficients, R^2^, RMSE, and FAC2. The values of these metrics demonstrated a strong association between predicted and observed concentrations and outperformed the counterpart multiple linear regression model. This paper emphasizes the use of these models, which can analyze the whole distribution of the pollutants, including both tails of the distribution, which are important from a public health perspective. Furthermore, it emphasizes that in addition to emission sources, meteorological parameters play an important role in controlling the levels of air pollution in urban areas, which should be considered when preparing an air quality management plan, particularly in hot climatic conditions, such as Jeddah.

This paper provides a robust methodology for O_3_ and NO_2_ modeling with improved model performance; however, the study overall has several limitations, which should be considered: (a) The results of this study are based on data from only one air quality monitoring station. To better aid public health planning and air quality management strategy, future work should aim to use data from considerably more monitoring stations over a wide area, possibly with diverse emission sources. (b) Both models for NO_2_ and O_3_ have no solar radiation data, which is one of the weaknesses of this study. Given that past work shows how solar radiation affects both NO_2_ and O_3_ concentrations, future work should consider including this important meteorological parameter in their modeling. (c) In urban areas, both NO_2_ and O_3_ levels are closely linked with traffic composition and flow; therefore, traffic characteristics should be included in future models or described in detail.

## Figures and Tables

**Table 1:** List of instruments for ambient air quality monitoring with their specifications, calibration, and detection limits [42].

Analyzer	NO_x_	SO_2_	O_3_	CO
Model	APNA-370	APSA-370	APOA-370	APMA-370
Application	NO_2_, NO, NO_x_	SO_2_, H_2_S	O_3_	CO
Principle	Cross flow modulation, Chemiluminescence	UV fluorescence	Cross flow modulation, UV absorption	Cross flow modulation, non-dispersive IR absorption
Range (ppm)	0–10	0–10	0–10	0–100
Lower Detectable limit (LDL)	0.5 ppb (3 sigma)	0.5 ppb (3 sigma)	0.5 ppb (3 sigma)	0.02 ppm (3 sigma)
Repeatability	±1.0% of F. S.	±1.0% of F. S.	±1.0% of F. S.	±1.0% of F. S.
Linearity	±1.0% of F. S.	±1.0% of F. S.	±1.0% of F. S.	±1.0% of F. S.
Zero drift (at lowest range)	<LDL/day ±1.0 ppb/week	<LDL/day <LDL/week	<LDL/day <LDL/week	<LDL/day <0.2 ppm/week
Span drift (at lowest range)	<LDL/day ±1.0% of F. S./week	<LDL/day <LDL/week	<LDL/day <LDL/week	<LDL/day ±1.0% of F. S./week
Response time (T_90_) (s) (at lowest range)	Within 90 s	Within 120 s	Within 75 s	Within 50 s
Sample gas				
flow rate (L/min)	0.8	0.7	0.7	1.5

**Table 2:** Summary of the air pollutants and meteorology data for 2018–2019. All of the pollutants were measured in ppb, except for CO, which was measured in ppm. In the table, WS stands for wind speed (m/s), WD for wind direction (degree from the north), Temp for temperature (°C), and RH for relative humidity (%).

	Metric	Min	1st Qu	Med	Mean	3rd Qu	Max	SD
Variable								
NO		0.01	2.04	5.43	13.5	13.92	146.36	21.57
NO_2_		0.82	17.84	26.60	28.25	37.97	93.39	13.29
NO_x_		1.32	21.85	32.86	41.74	51.02	197.34	30.80
O_3_		0	13.86	35.91	36.31	55.24	122.94	25.11
SO_2_		0	1.31	4.74	13.19	15.20	194.07	21.14
CO		0.03	0.18	0.26	0.32	0.37	2.38	0.23
WS		0	0.65	1.27	1.46	2.07	7.20	0.97
WD		0.84	251.61	336.63	292.39	340.96	350.20	78.19
Temp		18.98	27.19	30.61	30.47	33.80	47.74	4.45
RH		6.44	39.74	54.16	52.69	66.13	98.50	17.02

**Table 3:** Model coefficients at different quantiles (tau) of O_3_ concentrations.

	Tau	0.05	0.25	0.50	0.75	0.95
Parameter						
Intercept		24.29	20.65	14.61	13.02	14.50
NO		−0.27	−0.20	−0.21	−0.24	−0.22
NO_2_		−0.91	−1.12	−1.19	−1.12	−1.15
CO		21.41	19.91	22.68	21.99	23.37
SO_2_		0.05	0.06	0.10	0.15	0.45
WS		1.40	2.15	2.34	2.31	1.56
WD		0.04	0.04	0.04	0.02	0.01
Temp		0.28	1.00	1.62	2.14	2.72
RH		−0.19	−0.21	−0.22	−0.23	−0.25

**Table 4:** Model coefficients at different quantiles (tau) of NO_2_.

	Tau	0.05	0.25	0.50	0.75	0.95
Parameter						
(Intercept)		12.69	11.70	13.25	20.17	26.54
NO		−0.22	−0.15	−0.08	−0.08	−0.05
O_3_		−0.24	−0.30	−0.31	−0.30	−0.24
CO		40.89	35.34	30.58	34.55	41.26
SO_2_		0.10	0.09	0.09	0.10	0.08
WS		−0.52	−1.07	−1.77	−2.51	−3.63
WD		0.02	0.03	0.03	0.03	0.03
Temp		0.03	0.25	0.41	0.39	0.34
RH		−0.03	−0.01	−0.02	−0.03	−0.05

**Table 5:** Statistical metrics for cross-validated models using 25% of the randomly selected testing dataset. The values outside of the parenthesis are for quantile regression (QRM) and inside are for multiple linear regression model (MLRM).

Metrics	O_3_ QRM (MLRM)	NO_2_ QRM (MLRM)
Correlation Coefficient (r)	0.92 (0.78)	0.91 (0.81)
Coefficient of determination (R2)	0.86 (0.61)	0.83 (0.66)
RMSE (ppb)	14.42 (15.13)	8.96 (7.97)
FAC2	0.79 (0.73)	0.96 (0.91)

## Data Availability

The data can be available on appropriate request.
